# Ischemic preconditioning attenuates lipid peroxidation and apoptosis in the cecal ligation and puncture model of sepsis

**DOI:** 10.3892/etm.2013.1034

**Published:** 2013-04-02

**Authors:** ÇIMEN GÜLBEN OLGUNER, UĞUR KOCA, EMEL ALTEKIN, BEKIR UĞUR ERGÜR, SEDEN DURU, PELIN GIRGIN, AYDIN TAŞDÖĞEN, KERIM GÜNDÜZ, SEDA GÜZELDAĞ, MUHAMMED AKKUŞ, SERAP CILAKER MICILI

**Affiliations:** 1 Departments of Anaesthesiology and Reanimation, School of Medicine, Dokuz Eylül University, İzmir 35340;; 2 Biochemistry and Clinical Biochemistry, School of Medicine, Dokuz Eylül University, İzmir 35340;; 3 Histology and Embryology, School of Medicine, Dokuz Eylül University, İzmir 35340;; 4 Department of Anaesthesiology and Reanimation, Şifa Hospital, İzmir 35100;; 5 Department of Anaesthesiology and Reanimation, Samandağ State Hospital, Hatay 31800, Turkey

**Keywords:** ischemic preconditioning, lipid peroxidation, apoptosis, lung, kidney, sepsis

## Abstract

Sepsis and septic shock are are among the major causes of mortality in intensive care units. The lung and kidney are the organs most affected by sepsis. Evidence exists that lipid peroxidation and apoptosis may be responsible for the high mortality due to sepsis. Ischemic preconditioning (IP) is a method for the protection of tissues and organs against ischemia/reperfusion injury by reducing reactive oxygen species levels, lipid peroxidation and apoptosis. In the present study, the effects of IP were investigated in cecal ligation and puncture (CLP)-induced sepsis in rats. The three groups of animals used in the present controlled study were the sham-operated group (sham, n=7), which only underwent a laparotomy; the sepsis group (sepsis, n=7), which underwent cecal ligation and perforation; and the IP + sepsis group (IP+sepsis, n=7), which underwent CLP immediately prior to the application of three cycles of IP to the hind limb. The study was terminated at 6 h after the induction of CLP. Blood, kidney and lung tissue samples were collected for the determination of serum creatinine, blood urea nitrogen (BUN), neutrophil gelatinase-associated lipocalin (NGAL) and lung tissue malondialdehyde (MDA) levels, as well as histological examination. The serum creatinine, plasma NGAL and lung tissue MDA levels in the sepsis group were significantly increased compared with those in the sham and the IP+sepsis groups (P<0.05). Alveolar macrophage counts, histological kidney and lung injury scores, kidney (caspase 3) and lung tissue immuonreactivity (M30) scores in the sepsis group were also significantly increased compared with those in the sham and IP+sepsis groups (P<0.05). The alveolar macrophage count in the IP+sepsis group was increased compared with that in the sham group (P<0.05). In conclusion, IP inhibits lipid peroxidation and attenuates histological injury and apoptosis in the lung and kidney during sepsis.

## Introduction

Severe sepsis is a major cause of mortality in the intensive care unit (ICU). Despite advances in supportive care and diagnostic tools, severe sepsis and septic shock remain associated with high mortality rates (25–54%) (1–3). Although the induction of the inflammatory c–ascade in sepsis is mainly associated with infectious agents, it exhibits great similarity to ischemia/reperfusion (IR) injury in tissues. In particular, the free oxygen radicals that are generated during ischemia and the superoxide radicals that emerge during reperfusion cause endothelial injury, increased capillary permeability and tissue edema. The cytokines and adhesion molecules activated during IR initiate systemic inflammatory response syndrome (SIRS) which is also observed in sepsis (4–6).

Sepsis is one of the major extrapulmonary causes of acute lung injury (ALI) and acute respiratory distress syndrome (ARDS) (3). The pulmonary endothelium becomes lined with cytokine-containing proteins as a result of cytokine release, neutrophil activation and increased capillary permeability and reactive oxygen species (ROS) generation. ROS production at levels greater than the antioxidant capacity causes lipid peroxidation and consequently tissue and cell injury (7–9).

Another organ affected by sepsis is the kidney. Sepsis/septic shock causes acute kidney injury (AKI) via the modulation of renal inflammation by specific components of the sepsis-induced inflammatory cascade. Tubular cell apoptosis in the kidney may be significant in septic AKI (10,11).

Ischemic preconditioning (IP) consists of brief ischemic periods that prepare the tissue or organs for a subsequent long period of ischemia. The excitement of the endogenous protective mechanisms against ischemia is the basis of IP. This phenomenon was described by Murry *et al* for the first time in 1986 (12). The protective molecular or cellular mechanisms and mediators of IP remain unclear. However, there is much evidence that IP modulates adenosine receptors, activates K-ATP channels, decreases ROS production and reduces the expression of TNFα and NFκB (13–15).

Remote IP is another method of conducting IP. The basic mechanism of remote IP is preparing one organ for ischemic insult by the application of IP to another site of the body (generally a lower limb) (16–18). Limb IP decreases the IR injuries in the lung, liver, myocardium and kidney and ameliorates the hemodynamic response to tourniquet application (19–27). These previous studies have shown that limb IP is a promising method for the prevention of IR-induced tissue injuries in certain clinical conditions. However, the effect of limb IP on sepsis-induced tissue injury has not yet been extensively studied. In only one study, the inflammatory response was shown to be modulated by intestinal IP in an endotoxemic shock model (28).

The cecal ligation and puncture (CLP) rodent model is widely used as it mimics human polymicrobial sepsis more closely than lipopolysaccharide (LPS) administration. According to evidence-based data, CLP creates an inflammatory response after inducing sepsis, by increasing TNFα, IL-8 and ROS production, initiating apoptosis, decreasing mean arterial pressure and increasing lactate production (29–33)., However, limb IP appears to be a more feasible method for the treatment of the clinical conditions of patients with sepsis. Thus, we designed an experimental study using the CLP sepsis model in rats to evaluate the effects of limb IP on histological and biochemical parameters in the lung and kidney.

## Materials and methods

### 

#### Animals

A total of 21 adult male Wistar rats (body weight, 200–250 g) were used in the experiment. All animals were obtained from the Multidisciplinary Experimental Animal Laboratory at Dokuz Eylül University, School of Medicine, İzmir with the approval of the Animal Experiment Ethical Committee of Dokuz Eylül University, School of Medicine (İzmir, Turkey).

Animals were caged in groups of five with free access to food and water and were maintained on a 12-h light-dark cycle at a room temperature of 22±1°C.

The rats were anesthetized with a mixture of 50 mg/kg ketamine (Ketalar^®^, Pfizer Pharma GmbH, Berlin, Germany) and 10 mg/kg xylazine hydrochloride (Alfazyne^®^, 2%; Alfasan International, Woerden, The Netherlands) that was administered intraperitoneally. The doses were repeated for the immobilization of the rats while maintaining spontaneous ventilation.

#### Experimental sepsis model by CLP

The rats were subjected to CLP as previously described (34,35). Briefly, under aseptic conditions, a 3-cm midline laparotomy was performed to allow the exposure of the cecum and adjoining intestine. The cecum was tightly ligated with a 2.0-silk suture at its base, below the ileocecal valve, and was perforated twice with an 18-gauge needle. The cecum was then gently squeezed to extrude a small amount of feces from the puncture site. The cecum was then returned to the peritoneal cavity and the laparotomy was closed with 3.0-silk sutures. Sham-operated animals underwent the same surgical procedure although the cecum was neither ligated nor punctured. Saline (3 ml/100 g) was administered to all rats intraperitoneally at the end of the procedure. All animals were returned to their cages with free access to food and water.

#### IP

Left-lower-limb IP was performed by applying a rubber band tourniquet high around the left thigh for 10 min followed by reperfusion for 10 min, as previously reported. The cessation of the blood flow during the ischemic period was confirmed with laser Doppler flowmetry. Three cycles of limb IP were performed to achieve effective pre-conditioning (19).

#### Experimental groups and protocol

The three groups of animals used in the present study were the sham-operated group (sham, n=7), which underwent a laparotomy; the sepsis group (sepsis, n=7), which underwent CLP; and the IP-treated group (IP+sepsis, n=7), which underwent CLP immediately prior to the application of three cycles of IP.

The rats were kept at a constant environmental temperature of 37°C to maintain body heat following the procedures. At 6 h after the CLP, the rats were reanesthetized with the same dose of ketamine and xylazine hydrochloride, then their abdomens were opened and kidneys removed. Following a midline sternotomy, the rats were exsanguinated by needle aspiration of the right ventricle and their lungs were removed. Plasma samples and the left lungs were immediately transferred to a biochemistry laboratory and stored at −80°C for the determination of serum creatinine, blood urea nitrogen (BUN), plasma neutrophil gelatinase-associated lipocalin (NGAL) and lung tissue malondialdehyde (MDA) levels. The right lungs and kidneys were fixed in 10% formalin solution for histomorphological determination and apoptosis (cytokeratin 18 with M30 immunostaining for lungs and caspase-3 immunostaining for kidneys).

#### Serum creatinine and BUN levels

Serum creatinine and BUN were measured with a is urine/CSF protein clinical chemistry kit (Abbott-Laboratories, Abbott Park, IL, USA) on an Architect 16000 analyzer (Abbott Laboratories, Chicago, IL, USA). BUN and serum creatinine results are expressed as mg/dl.

#### Detection of lung tissue lipid peroxidation

Tissue homogenates were prepared by the mechanical disruption of tissue samples using a TissueLyser (Qiagen, Hilden, Germany) for 5 min at 30 Hz in 0.1 M phosphate buffer, pH 7.5. Tissue homogenates were then centrifuged at 10,000 × g, 4°C for 5 min. The upper clear supernatants were transferred to a 2-ml Eppendorf tube. The protein levels of the tissue samples were measured with a quantitative kit using an Architect 16000 analyzer (Abbott Laboratories). The concentrations of MDA in the samples were determined using a high-pressure liquid chromatography method as described by Hong *et al*(36), using a 5 *μ*M C-18 reversed-phase column (250 × 4.6 mm I.D) and a mobile phase of KH_2_PO_4_ (0.01 M) and 30% methanol with a fluorescent detector. The MDA results are expressed in *μ*mol/g protein.

#### Serum neutrophil gelatinase-associated lipocalin

Serum NGAL levels were quantified using a commercially available NGAL ELISA kit (Boster Biological Technology, Wuhan, China) according to the manufacturer’s instructions. The plasma NGAL results are expressed in pg/ml.

### Histomorphological procedures

#### Light microscopic tissue preparation

The tissue samples were fixed in 10% formalin in phosphate buffer for 24 h, then processed by routine histological methods and embedded in paraffin blocks. Sections of 4–5 *μ*m thickness were obtained and stained with hematoxylin and eosin (H&E), periodic acid-Schiff (PAS) and Masson’s trichrome stains. The H&E-stained sections were used to evaluate the general morphology; the PAS stain for the basal membrane and brush border of the tubules; and the Masson’s trichrome stain for the collagen content of the parenchyma. All histomorphological and immunohistochemical assessments of the lungs and kidneys were evaluated by two histologists blinded to the groups.

#### Histomorphological assessment of lung tissue

Digital images were obtained from the H&E- and Masson’s trichrome-stained sections using a digital camera (DP71; Olympus, Tokyo, Japan) connected to a light microscope (Olympus BX51). Three non-overlapping lung sections and a minimum of 30 lung fields were examined per animal. A grading system was used to score the general alveolar and parenchymal morphological changes, including alveolar structure, inflammation, thickening of the alveolar septum, alveolar macrophages, neutrophils, increased capillary permeability, hemorrhage, edema and congestion. The grading system was scored according to these findings by the absence (score, 0), or presence (score, 1 for mild, 2 for moderate, 3 for marked and 4 for diffuse) of these changes in the alveolar tissue (37).

The number of alveolar macrophages in the alveolar septum and lumen was visualized digitally and counted in a given area (macrophage count/0.016 mm^2^).

#### Histomorphological evaluation of kidney tissue

The sections were stained with H&E and PAS stains. Three non-overlapping kidney sections and a minimum of 30 kidney fields were examined per animal. The structural changes in the kidney tissue sections were evaluated using light microscopy and scored for proximal tubule damage (tubular atrophy, tubular brush border loss, vacuolization, tubular dilation and cast formation), mononuclear cell infiltration, erythrocyte extravasation, interstitial structural changes, renal corpuscle morphology and necrotic and apoptotic cells. The tubulointerstitial damage in the obtained cross-sectional images was scored semiquantitatively. The scoring system for these findings was: 0, none; 1, 1–25%; 2, 26–50%; 3, 51–75%; and 4, 76–100% (30).

#### Determination of apoptosis by immunohistochemical study

To detect DNA fragmentation in epithelial cells in the lung tissue, M30 with cytokeratin-18 was used, while active caspase-3 immunohistochemistry was used to evaluate apoptosis in the kidney tissue in the paraffin sections. Following routine immunohistochemical procedures, the sections were incubated overnight at 4°C with rat-specific anti-M30 antibody (1:100, SC-32329; Santa Cruz Biotechnology, Inc., Santa Cruz, CA, USA) and active form anti-caspase-3 antibody (1:100, AB3623; Millipore, Temecula, CA, USA). The sections were then stained with DAB and counterstained with Mayer’s hematoxylin. A grading system was used to score the quantity of anti-M30 and anti-caspase-3 positive staining in the sections (38). The score was defined as follows: 0, no immunoreactivity; 1, little positive staining; 2, moderate positive staining between grade 1 and grade 3; 3, marked positive staining evenly distributed across the whole image.

#### Statistical analysis

The Statistical Package for the Social Sciences 15 (SPSS 15.0; SPSS, Inc., Chicago, IL, USA) software was used for statistical analysis. Kruskal-Wallis and post hoc Mann-Whitney U tests were used for the comparison of biochemical results. The Chi-square test was used for comparison of the histopathological results. P<0.05 was considered to indicate a statistically significant difference. The results are expressed as the mean ± SD.

## Results

### Evaluation of the kidney

#### Biochemical results

The serum creatinine and BUN levels were significantly higher in the sepsis group compared with those in the IP+sepsis and sham groups (P<0.05). No significant differences were observed between the IP+sepsis and the sham groups (Table I).

**Figure 1:**
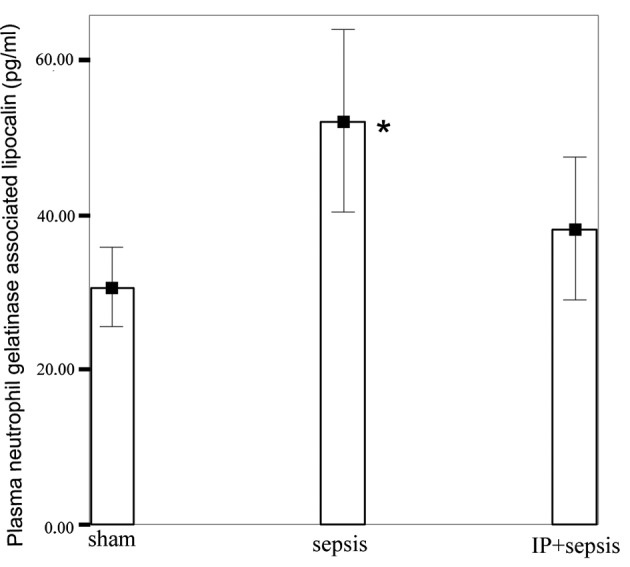
Plasma neutrophil gelatinase-associated lipocalin levels in all experimental groups. ^*^P<0.05 compared with the sham and IP+sepsis groups. IP, ischemic preconditioning.

NGAL levels in the sepsis group (52.05±11.67 pg/ml) were significantly higher than those in the sham (30.66±5.06 pg/ml) and IP+sepsis (38.17±9.15 pg/ml) groups (P<0.05; Fig. 1). No significant differences were observed between the sham and IP+sepsis groups.

#### Histomorphological and immunohistochemical results

The histological kidney injury and kidney tissue immuonreacimmuonreactivity (caspase 3) scores in the sepsis group were significantly increased compared with those in the sham and IP+sepsis groups (P<0.05). The kidney injury and the kidney tissue immunoreactivity (caspase 3) scores in the IP+sepsis group were not significantly different from those in the sham group (P>0.05; Table I).

#### Kidney photomicrographs

**Figure 2:**
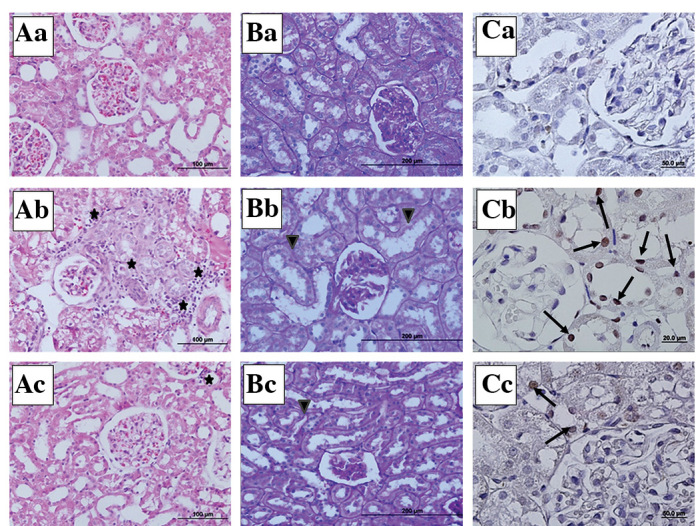
Representative micrographs of kidney tissue sections stained histochemically and immunohistochemically (H&E, PAS and active caspase-3 staining). (A) H&E (magnification, ×20), (B) PAS (magnification, ×10) and (C) active caspase-3 immunohistochemically stained sections (magnification, ×40) from the (a) sham, (b) sepsis and (c) IP+sepsis groups. ✶, mononuclear cell infiltration; ▾, irregular basal membrane and brush border loss in proximal tubular epithelium; →, active caspase-3 immunpositive cells; H&E, hematoxylin and eosin; PAS, periodic acid-Schiff; IP, ischemic preconditioning.

In the stained kidney tissue sections, the sham group showed a normal kidney structure (Fig. 2Aa,Ba and Ca). In the sepsis group, mononuclear cell infiltration around the glomeruli and capillaries, vasodilation, scattered tubular degeneration and cast formation in the tubules were observed (Fig. 2Ab). In the PAS-stained sections, loss of the brush border and irregularity in the basal membrane were noted in the proximal tubular cells of the sepsis group (Fig. 2Bb). In the IP+sepsis group, the histomorphological changes were less evident compared with those in the sepsis group (Fig. 2Ac and Bc). In the active caspase-3 immunohistochemical staining, the number of caspase-3 immunopositive cells was higher in the tissue sections of the sepsis group than in those of the sham and IP+sepsis groups (Fig. 2Cb and Cc).

### Evaluation of the lung

#### Determination of lipid peroxidation

**Figure 3:**
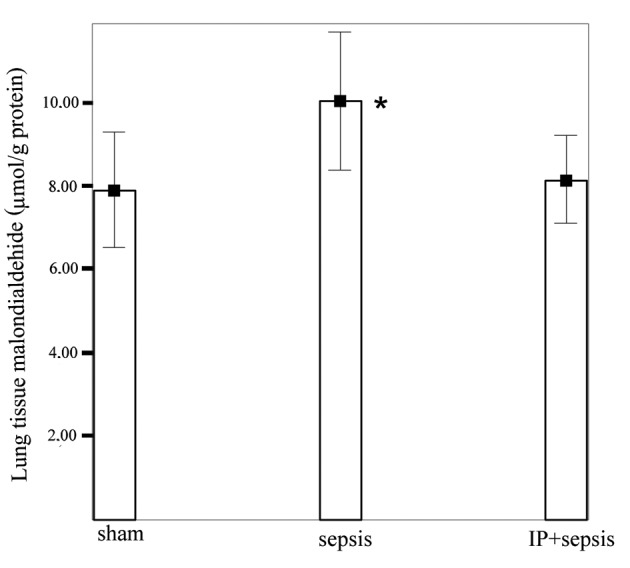
Lung tissue malondialdehyde levels in all experimental groups. ^*^P<0.05 compared with the sham and IP+sepsis groups. IP, ischemic preconditioning.

The lung tissue MDA levels were increased significantly in the sepsis group (10.02±1.66 *μ*mol/g protein) compared with those in the sham (7.90±1.39 *μ*mol/g protein) and IP+sepsis (8.14±1.05 *μ*mol/g protein) groups (P<0.05). No significant differences were detected between the IP+sepsis and sham groups (Fig. 3).

#### Histomorphological and immunohistochemical results

**Figure 4:**
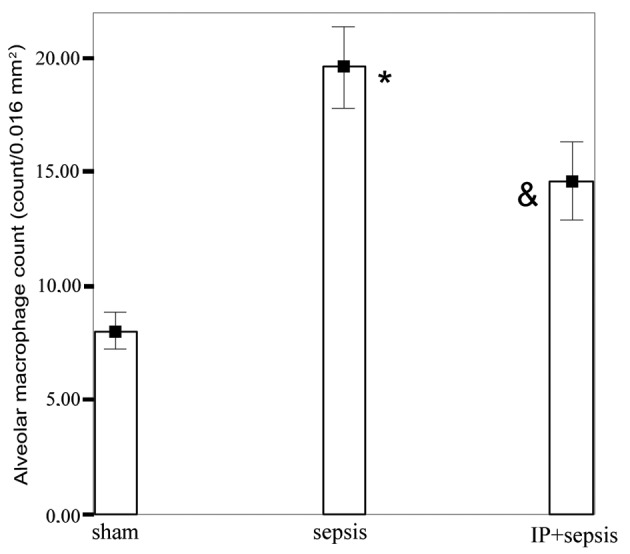
Alveolar macrophage count in all experimental groups. ^*^P<0.05 compared with the sham and IP+sepsis groups. ^&^P<0.05 compared with the sham group. IP, ischemic preconditioning.

The histological lung injury and lung tissue immunoreacimmunoreactivity (M30) scores in the sepsis group were significantly increased compared with those in the sham and IP+sepsis groups (P<0.05). There were no differences in the lung tissue immunoreactivity (M30) scores between the IP+sepsis and sham groups. The lung injury score in the IP+sepsis group was elevated compared with that in the sham group (Table II). The alveolar macrophage count in the sepsis group (19.57±1.81) was significantly increased compared with those in the sham (8.00±0.81) and IP+sepsis (14.57±1.71) groups (P<0.05) and was higher in the IP+sepsis group than in the sham group (P<0.05; Fig. 4).

#### Lung photomicrographs

**Figure 5:**
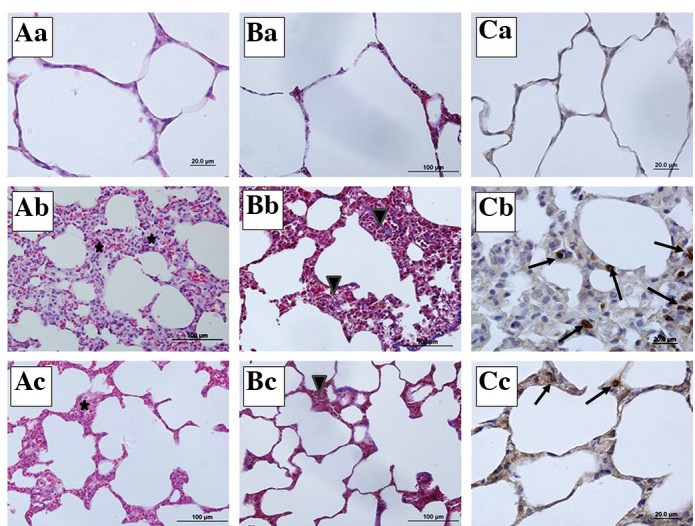
Representative micrographs of lung tissue sections stained histochemically and immunohistochemically (H&E, Masson’s trichrome and M30 staining). (A) H&E (magnification, ×40), (B) Masson’s trichrome (magnification, ×20), (C) M30 immunohistochemically stained sections (magnification, ×20) from the (a) sham, (b) sepsis and (c) IP+sepsis groups. ✶, mononuclear cell infiltration; ▾, collagen content of the parenchyma; →, marked M30 immunopositive cells; H&E, hematoxylin and eosin; IP, ischemic preconditioning.

The staining of the lung tissue sections showed that the sham group had normal lung histology (Fig. 5Aa, Bb and Cc). By contrast, there was a significant increase in the lung injury score following sepsis, with the development of alveolar wall thickening and inflammatory infiltration (Fig. 5Ab and Bb). When the sections from the sepsis group were observed, inflammation and thickening of the alveolar septum, particularly increases in alveolar macrophage and mononuclear-cell infiltration which ensured that the alveolar septum increased capillary permeability, hemorrhage, edema and congestion, were noted (Fig. 5Bb). However, in the IP+sepsis group, the structural lung injury was less severe compared with that in the sepsis group (Fig. 5Ac and Bc). In the Masson’s trichrome-stained sections, increased collagen content in the parenchyma of the lung tissue sections of the sepsis group was observed (Fig. 5Bb). Furthermore, in the M30 immunohistochemistry scoring, the number of M30 immunopositive epithelial cells was increased in the sepsis group compared with the numbers in the sham and IP+sepsis groups (Fig. 5Cb and Cc).

## Discussion

In the present study, it was shown histologically and biochemically that IP applied to the unilateral hind limb attenuated septic lung and kidney injury. To the best of the author’s knowledge, the present study is the first to evaluate remote IP with regard to lung and kidney injury in a CLP sepsis model. CLP was selected for the generation of sepsis as it is a widely used experimental sepsis model that mimics human polymicrobial sepsis (34,39).

Experimental and clinical studies have shown that IP increases resistance against ischemic tissue injury to the organs, including the heart, lung, kidney, intestine, liver and muscle (20,25,28,40,41). SIRS may be induced by infection as well as IR. The mechanisms by which infections and IR trigger SIRS are almost the same. Therefore, it has been hypothesized that either ischemic or pharmacologically-simulated preconditioning may lead to new possibilities in the treatment of critically ill patients with sepsis and multiple organ dysfunction syndrome (MODS) (4,24).

The main ischemic conditioning methods are preconditioning (prior to major ischemia), perconditioning (during major ischemia) and postconditioning (during reperfusion) which are named according to the application time (42). In the present study, IP was initiated immediately following the CLP procedure and completed within 1 h. It has been shown that sepsis occurs 5 h after CLP (35,43). Therefore the present method of ischemic conditioning was described as ischemic ‘preconditioning’. The effectiveness of IP is associated with the number of ischemic cycles and length of the ischemic period (17,44). In the present study, three 10 min IP cycles were used, for which effectiveness against IR had already been demonstrated (19), and the blood and tissue samples were collected 6 h after the CLP procedure.

The kidney is the one of the main organs affected during sepsis. Decreased renal perfusion and numbers of cortical capillaries, peaking of renal tubular apoptosis and increased leukocyte infiltration and ROS production have been observed in experimental models 4–6 h after the induction of sepsis (29,32,45,46).

The protective effects of limb IP are transferred by means of heat shock proteins, nitric oxide, adenosine, bradykinin and neurogenic signals to the target organs (13,47). The results of these interactions are reductions in ROS levels, reductions in ATP depletion and increases in ATP production which consequently increase the oxidative stress resistance of the cell (13,15). The effect of limb IP on IR injury to the kidney has been shown to reduce serum creatinine and BUN levels (48). Er *et al*(49) and Zimmerman *et al*(25) demonstrated similar results in clinical studies. In the present study, it was observed that the BUN and serum creatinine levels were significantly higher in the sepsis group than in the IP+sepsis group, while no significant differences were observed between the IP+sepsis and sham groups. These findings indicate that IP protects renal function from sepsis-related kidney insult. This effect of limb IP may be associated with the reduction of ROS production or an increase in microvascular circulation.

NGAL is a highly sensitive, specific and predictive early biomarker for AKI (50,51). Bagshaw *et al*(52) observed that patients with acute kidney sepsis have higher plasma and urine NGAL levels compared with patients without acute septic kidney injury. Furthermore, serum NGAL levels have been observed to be elevated in SIRS, sepsis and septic shock (53,54). NGAL is present in the kidney, liver, spleen, lung and trachea, indicating that serum NGAL may not be a reliable marker of septic AKI (55). By contrast, Chen *et al*(14) showed that a major cause of elevated serum NGAL in the kidney is ischemic renal injury and the application of IP to the kidney decreases NGAL levels. Therefore, it may be concluded that the elevation of the plasma NGAL level in the present sepsis group may be associated not only with the kidney, but also other NGAL-containing tissues such as the lung, spleen and liver. However, the increase in the plasma NGAL level of the sepsis group reflected the sepsis-induced NGAL release from tissues which occurred in the present study. The present study also showed that the sepsis-associated NGAL release from tissues was efficiently inhibited by limb IP.

In sepsis, neutrophil accumulation in the lung is followed by cytokine and ROS production. ROS production exceeding the antioxidant capacity causes lipid peroxidation and consequent tissue injury and cell death (9,56). MDA is the end-product of fatty acid peroxidation and is generated within inflammatory cells. The MDA content of the lungs increases significantly following CLP-induced sepsis (43,57,58). Numerous studies have shown that IP diminishes neutrophil accumulation, lipid peroxidation and MDA levels in the lung (19,23,59–61). In the present study, the lung MDA levels were significantly higher in the sepsis group than in the IP+sepsis and sham groups. We conclude that remote IP has an antioxidant effect in CLP-induced sepsis by decreasing lipid peroxidation levels in the lung

Previous studies have investigated lung and kidney injuries using H&E staining and light microscopy. Notably high histological lung and kidney injury scores have been observed in septic rats (62–65). IP attenuates lung and kidney histological injuries in ischemic conditions and Lee *et al*(66) histologically showed that hepatic IP is able to attenuate renal ischemic injury. It has been demonstrated that IP acts via A1 adenosine receptor activation (15,41). The activation of the A1 adenosine receptor diminishes renal inflammation, apoptosis and necrosis in renal ischemia (16,67).

We have previously demonstrated that unilateral hind limb IR causes lung injury and remote IP decreases the lung injury score (19). Similarly, lung injury has been mitigated using limb injury in the hemorrhagic shock model (5). In the present study it was observed that the lung and kidney histological injury scores were significantly lower in the IP+sepsis group than in the sepsis group. Therefore, we propose that IP reduces sepsis-induced lung and kidney injury not only biochemically, but also histologically.

The mononuclear phagocyte system is involved in phagocytosis and numerous complex immunological and inflammatory processes. Sepsis is associated with the increased production of cellular proinflammatory and inflammatory mediators by monocytes/macrophages. TNFα is the main mediator and has an important role in acute kidney and lung injury in sepsis (68,69). Remote IP decreases the levels of TNFα and other inflammatory cytokines, including IL-6 and IL-8, in lung tissue and plasma, (61,70). However, ROS activate the transcription factor NFκB which stimulates the excessive production of inflammatory cytokines. Takeshita *et al*(71) showed that direct IP reduced NFκB activity and cytokine mRNA levels.

In the present study, a significantly higher alveolar macrophage count was observed in the sepsis group than in the sham and IP+sepsis groups. We were unable to measure the cytokine levels, which was a limitation of the present study. However, the CLP-induced alveolar macrophage count in the lung was decreased by limb IP. This effect may be explained by the reduction of TNFα levels and the inhibition of NFκB activity.

Apoptosis is a type of programmed cell death in which DNA disintegration and cell death occur as a result of the activation of death-inducing receptors or intracellular specific serine proteases (caspases) (72,73).

The gold standard for the diagnosis of apoptosis is morphological/ultrastructural evaluation. The determination of apoptosis using H&E staining and light microscopy is sensitive and specific, while also being the least expensive approach (74,75). However, to evaluate acute injury such as in CLP-induced sepsis, more early markers that indicate apoptosis are required. Therefore, caspase 3 and M30 (caspase cleaved cytokeratin 18 neo-epitope) staining were used in the present study to show kidney and lung epithelial apoptosis, respectively. The sensitivity of these two methods has been demonstrated clinically and experimentally, including in CLP-induced sepsis (11,57,76–78).

Apoptosis occurs through inflammatory cytokines and TNFα, apoptosis-associated proteins and ROS-mediated pathways during sepsis (69,79–82).

IP reduces apoptosis by regulating the genes which encode these proteins and releasing the metabolites involved in the apoptotic process (83). The protective effect of IP on IR injury-induced apoptosis has been demonstrated in the lung and kidney (80,83).

In the present study, significant increases were observed in the M30-positive cell count of the sepsis group compared with the sham group, indicating the presence of epithelial cell-specific apoptosis in sepsis. Also, no significant changes were noted in the number of pulmonary M30-positive cells in the IP+sepsis group compared with the sham group, suggesting that limb IP inhibits apoptosis in CLP-induced sepsis. Additionally, it was also observed that number of caspase-3-stained renal tubular cells was significantly lower in the IP+sepsis group than in the sepsis group and not significantly different between the sham and IP+sepsis groups. Jacobs *et al*(10) suggested that intra-renal inflammation is decreased by caspase inhibition through unclear mechanisms. It is possible that IP inhibits caspase. Together, these results suggest that IP attenuates apoptosis in the kidney and the lung during sepsis.

In conclusion, the present study presents evidence for the effectiveness of IP in the management of sepsis. Remote IP was observed to reduce lung lipid peroxidation, kidney and lung injuries and apoptosis in a CLP-induced sepsis model. Further investigations are required to fully elucidate the underlying mechanisms of IP in sepsis.

## Figures and Tables

**Table I:** Serum creatinine, BUN, kidney injury and apoptosis (caspase 3) scores in all experimental groups.

Group	Serum creatinine (mg/dl)	BUN (mg/dl)	Kidney injury score (0–4)	Caspase 3 score (0–3)
Sham	0.50±0.22	21.14±3.62	0.28±0.48	0.42±0.53
Sepsis	0.58±0.05^a^	29.28±3.99^a^	1.71±0.48^a^	1.57±0.53^a^
IP+sepsis	0.52±0.03^b^	24.00±3.82^b^	0.57±0.78^b^	0.85±0.37^b^

All data are the mean ± SD (n:7).

a P<0.05 compared with the sham group;

b P<0.05 compared with the sepsis group. IP, ischemic preconditioning; BUN, blood urea nitrogen.

**Table II:** Histological lung injury and apoptosis (M30) scores in all experimental groups.

Group	Lung injury score (0–4)	M30 score (0–3)
Sham	0.14±0.37	0.42±0.53
Sepsis	2.00±0.57^ab^	2.00±0.57^ab^
IP+sepsis	0.71±0.71^a^	0.85±0.37

All data are the mean ± SD (n=7).

a P<0.05 compared with the sham group;

b P<0.05 compared with the IP+sepsis group. IP, ischemic preconditioning.
